# A Heuristically Accelerated Reinforcement Learning-Based Neurosurgical Path Planner

**DOI:** 10.34133/cbsystems.0026

**Published:** 2023-05-11

**Authors:** Guanglin Ji, Qian Gao, Tianwei Zhang, Lin Cao, Zhenglong Sun

**Affiliations:** ^1^ School of Science and Engineering, The Chinese University of Hong Kong, Shenzhen, China.; ^2^ Shenzhen Institute of Artificial Intelligence and Robotics for Society, Shenzhen, China.; ^3^ Department of Automatic Control and Systems Engineering, The University of Sheffield, UK.

## Abstract

The steerable needle becomes appealing in the neurosurgery intervention procedure because of its flexibility to bypass critical regions inside the brain; with proper path planning, it can also minimize the potential damage by setting constraints and optimizing the insertion path. Recently, reinforcement learning (RL)-based path planning algorithm has shown promising results in neurosurgery, but because of the trial and error mechanism, it can be computationally expensive and insecure with low training efficiency. In this paper, we propose a heuristically accelerated deep Q network (DQN) algorithm to safely preoperatively plan a needle insertion path in a neurosurgical environment. Furthermore, a fuzzy inference system is integrated into the framework as a balance of the heuristic policy and the RL algorithm. Simulations are conducted to test the proposed method in comparison to the traditional greedy heuristic searching algorithm and DQN algorithms. Tests showed promising results of our algorithm in saving over 50 training episodes, calculating path lengths of 0.35 after normalization, which is 0.61 and 0.39 for DQN and traditional greedy heuristic searching algorithm, respectively. Moreover, the maximum curvature during planning is reduced to 0.046 from 0.139 mm^−1^ using the proposed algorithm compared to DQN.

## Introduction

Keyhole neurosurgery is a procedure to insert the instrument deep inside the brain with predefined paths for biopsy and therapies in minimally invasive surgery for human brains. However, during the procedure, rigid instruments, such as traditional needle insertion might cause damage to anatomical obstacles such as blood vessels, which might lead to intraoperative cerebral hemorrhage in the worst case. The steerable needle has been implemented in various surgical operations [1–3] to perform complex curvilinear trajectories, for the benefit of its unique structure. Path planning of a steerable needle is a computational problem to find a sequence of waypoints from the entrance point to the target point and also avoid collisions with anatomical obstacles. Different from traditional rigid needles, the curved insertion path of a steerable needle is difficult to be planned manually by surgeons. Thus, an autonomous or semiautonomous path planner is required to safely operate the needle.

Various path planning algorithms have been proposed for steerable needles or catheters. They can be generally categorized into graph-based methods, geometric algorithms, artificial potential field (APF) methods, optimization-based algorithms, sampling-based methods, and learning-based methods [4]. Leibrandt et al. [5] proposed a graph-based rapid path planning algorithm for concentric tube robots by using parallel computation, which solved the time-consuming problem but could be computational resource intensive as well. In terms of the APF-based path planning algorithm, which is easy for the agent to be stuck in local minima, Zhao et al. [6] proposed a 2-dimensional (2D) steerable needle path planner using a modified APF algorithm in a planar environment with only several obstacles. Optimization-based algorithms, especially the particle swarm optimization algorithm, and evolutionary algorithm have been adopted a lot. An evolutionary optimization-based 3D path planning algorithm [7] was adopted in a programmable bevel-tip needle. Segato et al. [8] proposed a learning-based planning algorithm using the inductive learning and deductive reasoning framework, which can derive optimal insertion paths. However, their planning scheme is only suitable for programmable needles without need to consider rotations of the needle. They proposed an inverse RL-based (IRL) algorithm [9] for programmable needle path planning as well. The proposed IRL can give intraoperative planned paths to the users. However, both of the proposed algorithms required expert’s demonstrations, which were also time consuming but were not counted in the execution time. Furthermore, the IRL algorithm required an accurate model, which was not applicable.

Sampling-based algorithms prevent the discretization of maps that occurs in graph-based methods, leading to a important reduction in the computation time for large-scale maps. Xu et al. [10] proposed an Rapidly exploring Random Trees (RRT)-based motion planning method for a bevel-tip needle in 3D environments. However, the random exploration in the workspace makes a probabilistic trade-off between faster elapsed time and finer exploration, which means that the resulting path might not be the best solution. Aghdam and Liu [11] proposed a heuristic RRT-based multiple target path planner for 2D steerable needle insertion without having to completely retract and reinsert the needle for different targets. In the neurosurgery context, Caborni et al. [12] proposed a safety considerate path planner that provides relatively safe trajectories for neurosurgical needle intervention. However, the proposed method can only produce paths with discrete curvatures.

Reinforcement learning (RL) is a branch of machine learning that emphasizes an agent’s interaction with the environment to maximize the accumulated reward of the system [13]. Because of its capacity to generalize across diverse maps, RL has been applied in different path planning scenarios [14–16]. For steerable needles, Lee et al. [17] proposed a deep Q network (DQN)-based planning algorithm. However, the algorithm requires 2,000 training episodes to reach only one specific target tumor position from only one entry point, which is time consuming. Segato et al. [3] proposed a GPU-based Asynchronous Advantage Actor-Critic (GA3C) RL-based path planning scheme for the steerable catheter in the context of neurosurgery, which also suffers from poor training efficiency.

Considering the above 2D and 3D path planning of steerable needles, they all neglected the torsion effect. 2D needle-based path planning algorithms surely find paths with curvature constraints and neglect the influence of torsions [18] because there is no torsion in a planar trajectory. However, from the perspective of clinical neurosurgeries, the 3D insertion trajectories, with torsion considerations, are necessary for deep brain surgeries such as deep brain stimulation. When steering a bevel-tip needle in a 3D environment, the insertion trajectory with the smallest torsions is more applicable, because 3D trajectories with the smallest torsions result in the fewest changes of insertion planes during surgery. Torsion constraints have been widely implemented in needle control problems. A control framework [19] considered that torsion friction during needle insertion was proposed from the perspective of control. By combining a torsion model with the needle dynamics, a compensation strategy was proposed by Swensen and Cowan [20]. However, the proposed 3D path planner in needle-based neurosurgery [3,7,21] and all the mentioned needle-based path planning algorithms neglected the torsion in 3D trajectories.

In this paper, we propose a steerable needle preoperative safety–critical path planning algorithm based on a heuristically accelerated DQN (HADQN) with a fuzzy logic scheme as shown in Algorithm 1. The proposed algorithm is constrained by curvature and torsion energy functions as well. During the action selection stage of the DQN algorithm, the heuristic function is engaged. The purpose of the fuzzy logic scheme is to emphasize the safety criteria of the system. Promising results such as high training efficiency and minimal energy curves were shown in simulation experiments. The primary contributions are:

1. A novel HADQN-based preoperative path planning algorithm with curve energy-constrained heuristic policy for the nonholonomic steerable needle is proposed. The combined heuristic policy is constrained by the minimal curve energy function to minimize the curvature and torsion during planning. To the best of our knowledge, this is the first attempt of constrained a path with torsion energy in needle-based path planning.
2. A fuzzy inference system is proposed to balance the heuristic policy and the RL algorithm so that the balancing coefficient *λ* can be selected adaptively.
3. A fuzzy logic-based HADQN is proposed in this paper to safely plan the trajectory of the steerable needle insertion process. The proposed algorithm is compared to HADQN, DQN, and greedy heuristic searching (GHS) in training efficiency, path length, distance to obstacles, and maximum curvature, 4 aspects. Promising results can be seen in Discussion.

The rest of the paper is organized as follows. After introducing geometric, Markov decision process (MDP), HADQN preliminaries, the proposed FL-HADQN algorithm is described in Materials and Methods. As a comparison to DQN and GHS algorithms, the algorithm is trained in a human brain model and validated by executing the algorithm among 20 pairs of entry points and target points in Discussion.

## Materials and Methods

In this section, the proposed algorithm is explained from defining an MDP to combining heuristics and fuzzy inference system. The kinematics of a bevel-tip needle is given to represent the action space. A torsion and curvature equation are incorporated with the reward function. The overall combinations result in the proposed fuzzy logic-heuristically accelerated deep Q network (FL-HADQN) and the framework is illustrated in the “Heuristically accelerated deep Q network (HADQN)” section.

### Kinematics modeling and Markov decision process

RL issues can be expressed by solving the MDP. An MDP solver can give an optimal sequence of state transitions in the workspace. At each transition, a reward is given back to the agent to evaluate the action performance. The basic MDP can be represented by ⟨*S*, *A*, *P*, *R*, *γ*⟩. Each state *s_i_* in state space *S* is denoted by (*x*_tar_ − *x_i_*, *y*_tar_ − *y_i_*, *z*_tar_ − *z_i_*). *x_i_*, *y_i_*, and *z_i_* are the needle tip position along *X*, *Y*, and *Z* axis, respectively, while *x*_tar_, *y*_tar_, and *z*_tar_ are the target coordinates.

To derive the action space *A*, the steerable needle kinematics is given first. The motion of a bevel-tip needle (Fig. 2) relies on the interaction of the bevel-tip and tissue where a contact force is perpendicularly applied on the surface of the bevel tip. Consider inserting a bevel-tip needle into a soft tissue, contact forces, frictions, and torsions would be applied to the whole body of the needle, which makes the needle kinematics and dynamics modeling difficult. All the forces on the needle result in bendable trajectories with certain curvatures.

Actions are selected to rotate the needle tip local frame from 0° to 360°. The rotation consists of 8 different actions, which are 45° from each other. The insertion arc *l* is considered to be a straight line in a discrete format and, to be specific, is (0.1,0.2,0.3). Different *l* achieves variable curvatures during planning. The total action in one step can be described as (rotation, arc length). There are 8 rotation actions and 3 arc length actions; thus, a total of 24 actions are in the action space *A*.

*P* stands for state transition probability from *s* ∈ *S* to *s*′ ∈ *S* when taking action *a* ∈ *A*. The reward *R* is a feedback value to the agent during interaction with the environment, which is one of the most important factors in RL issues. Details of the reward function are discussed in the following subsection. The last factor *γ* is the discount factor of the training process that is related to caring more about the future or the current reward. If *γ* = 0, then the algorithm would be totally a greedy process that only cares about the current reward. Thus, to consider an optimal solution, *γ* should be as close to one as possible.

### Path formulation and reward functions

**Fig. 1.**
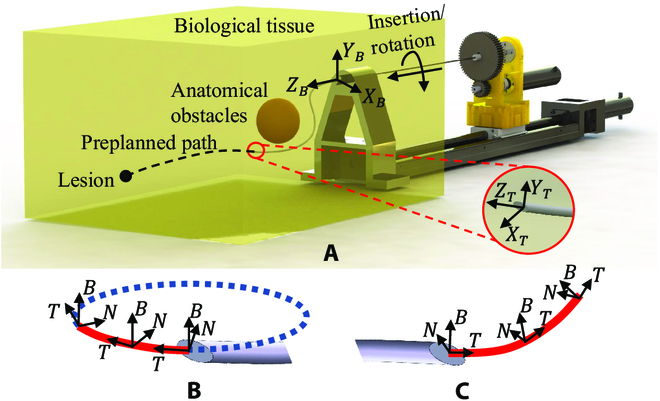
(A) Needle insertion system for neurosurgery. The lesion is the target point of the insertion task; the preplanned path is the path derived by the proposed algorithm; the anatomical obstacles are blood vessels; *X_B_*, *Y_B_*, and *Z_B_* are *x*, *y*, and *z* axes in base frame, respectively; *X_T_*, *Y_T_*, and *Z_T_* are *x*, *y*, and *z* axes in tip frame, respectively. (B) Frenet frame of a planar trajectory. (C) Frenet frame of a spatial trajectory.

The planned paths are formulated by discrete points and smoothed by the Bezier curve interpolation algorithm. The state *s_i_* = (*x*_tar_ − *x_i_*, *y*_tar_ − *y_i_*, *z*_tar_ − *z_i_*) of the RL agent represents the Euclidean distance between the tip position and the target position. To form paths by selecting the best action in each state, a reward function is that, by maximizing the designed reward functions described in this section, certain states are derived to form the planned trajectory,

$$R\left(s_{n}\right)=-10\cdot d_{1}\left(s_{n}\right)+d_{2}\left(s_{n}\right)+\|c\left(s_{n}\right)\|$$ (1)

where *d*_1_(*s_n_*) represents the value of Euclidean distance between the needle tip and the target position; the closer the end effector to the target, the higher the value in the first term. The second term *d*_2_(*s_n_*) is a punishment for reaching obstacles. The last term **c**(*s_n_*) is the curvature and torsion constraints of the bevel-tip needle, which is constructed by the energy along each axis: *c_x_*(*s_n_*), *c_y_*(*s_n_*), *c_z_*(*s_n_*). The smaller curvature and torsion give a shorter path, fewer rotations, and a safer working condition for the bevel-tip needle. The energy of the curve could be expressed by the curve energy function, which constraints the curvature and torsion,

$$c\left(s_{n}\right)=\sum_{i=1}^{n}{\left(k\left(s_{i}\right)+\tau \left(s_{i}\right)\right)}^{2}r\left(s_{i}\right)$$ (2)

where **r**(*s_i_*) = 〈*x*(*s_i_*), *y*(*s_i_*), *z*(*s_i_*)〉 is the curve equation for the discrete waypoints at state *s_i_* and the curve energy **c**(*s_n_*). *k*(*s_i_*) and *τ*(*s_i_*) are the curvature and torsion of the *i_th_* segment of the path and can be calculated as below,

$$k\left(s_{i}\right)=\frac{\left\|r^{\prime }\left(s_{i}\right)\times r^{\prime \prime }\left(s_{i}\right)\right\|}{{\left\|r^{\prime }\left(s_{i}\right)\right\|}^{3}}$$ (3)

$$\tau \left(s_{i}\right)=-N\cdot B^{\prime }=\frac{\det\left(r^{\prime }\left(s_{i}\right),r^{\prime \prime }\left(s_{i}\right),r^{\prime \prime \prime }\left(s_{i}\right)\right)}{{\left\|r^{\prime }\left(s_{i}\right)\times \left(r^{\prime \prime }\left(s_{i}\right)\right)\right\|}^{2}}$$ (4)

where **N** and **B** represent the normal unit vector and binormal unit vector of the frenet frame as shown in Fig. 1. Then, the **r**′(*s_i_*) and **r**′′(*s_i_*) can be calculated by Taylor series expansions if and only if *i* ≤ *n* − 2,

$$\begin{array}{c} x\left(s_{i+1}\right)=\left(x\left(s_{i}\right)+{\mathrm{hx}}^{\prime }\left(s_{i}\right)+\frac{h^{2}}{2}x^{\prime \prime }\left(s_{i}\right)+o{\left(h\right)}^{3}\right)\\ x\left(s_{i-1}\right)=\left(x\left(s_{i}\right)-{\mathrm{hx}}^{\prime }\left(s_{i}\right)+\frac{h^{2}}{2}x^{\prime \prime }\left(s_{i}\right)+o{\left(h\right)}^{3}\right)\\ x^{\prime }\left(s_{i}\right)=\frac{x\left(s_{i+1}\right)-x\left(s_{i-1}\right)}{2h}\\ x^{\prime \prime }\left(s_{i}\right)=\frac{x\left(s_{i+1}\right)-2x\left(s_{i}\right)+x\left(s_{i-1}\right)}{h^{2}}\\ x^{\prime \prime \prime }\left(s_{i}\right)=\frac{x\left(s_{i+2}\right)-2x\left(s_{i+1}\right)+2x\left(s_{i-1}\right)-x\left(s_{i-2}\right)}{2h^{3}} \end{array}$$ (5)

where *h* is the distance between the derivative and the second derivative of *y*(*s_i_*) and *z*(*s_i_*) that can also be derived as above. **r**′(*s_i_*) = 〈*x*′(*s_i_*), *y*′(*s_i_*), *z*′(*s_i_*)〉, **r**′′(*s_i_*) = 〈*x*′′(*s_i_*), *y*′′(*s_i_*), *z*′′(*s_i_*)〉, and **r**′′′(*s_i_*) = 〈*x*′′′(*s_i_*), *y*′′′(*s_i_*), *z*′′′(*s_i_*)〉. Because when *i* > *n* − 2, *x*(*s_i_* + 2) does not exist; thus, we make *x*′(*s_n_* − 2) = *x*′(*s_n_* − 1) = *x*′(*s_n_*), *x*′′(*s_n_* − 2) = *x*′′(*s_n_* − 1) = *x*′′(*s_n_*), and *x*′′′(*s_n_* − 2) = *x*′′′(*s_n_* − 1) = *x*′′′(*s_n_*) so that the curve can be smooth enough. The above reward function is used in all the training processes. The generated trajectories with constraints and without constraints are compared in Discussion.

**Table:** 

**Algorithm 1.** HADQN algorithm.
**Input: Brain Model, Entry Point, Target Point** **Output: Optimal Path** 1: Initialization: Replay buffer *D*, action–value function *Q* withrandom weight *θ*, and target action–value function $\hat{Q}$ withrandom weight *θ*^−^ = *θ* 2: **for** episode = 1, 2…*M* **do** 3: Initialize state *s_t_* from entry point, *r_t_* = 0 4: **for** *t* = 1, 2, …, *T* **do** 5: Generate heuristic policy *π^H^*(*s_t_*) 6: Calculate heuristic function *H_t_*(*s_t_*, *a*) by Eq. 8 7: Derive *λ* from fuzzy inference system 8: Execute the combined policy *a_t_* = *π^C^* by Eq. 6 and observe reward *r_t_* and new state *s*_*t* + 1_ 9: Store the transition pair (*s_t_*, *a_t_*, *r_t_*, *s*_*t* + 1_) in *D* 10: Sample random minibatch of transitions (*s_j_*, *a_j_*, *r_j_*, *s*_*j* + 1_) from *D* 11: Calculate temporal difference target *y_j_* 12: Perform a gradient descent step on (*y_j_* − *Q*(*s_j_*, *a_j_*; *θ*))^2^ according to Eq. 10 13: Reset *θ*^−^ = *θ* every *C* steps 14: **end for** 15: **end for** 16: Path smoothing by Bezier curve interpolation

**Fig. 2.**
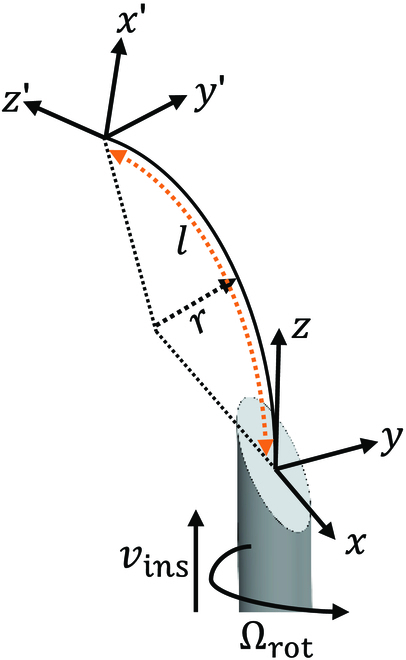
Geomatric representation of a circular segment of the needle insertion process from frame {*x*, *y*, *z*} to a new frame {*x*^′^, *y*^′^, *z*^′^}, where *l* is the insertion length at the current step, *r* = 1/*k*.

### Heuristics combination

Because the original DQN algorithm suffers from inefficient exploration in dense maps, a heuristic function is combined in the framework. The heuristic method for path planning is the most intuitive way to derive the policy at the current step; however, they are usually locally optimal instead of globally optimal. Thus, the heuristic policy is only to guide the agent to the target instead of utilizing this policy for the planner, which is helpful to training efficiency and also avoids local optimal solutions. By combining a heuristic policy, an HADQN algorithm is designed to solve the above MDP by influencing the choice of actions with a heuristic function *H_t_* that is designed to attract the trajectory toward the target such as the HAMMQ algorithm proposed by Bianchi et al. [22]; thus, a heuristically combined policy *π^C^* is given by,

$$\pi^{C}=\left\{\begin{array}{cc} {\text{argmax}}_{a}(Q_{1}(s,a)+\lambda H_{t}(s,a)), & p\geq \varepsilon \\ a_{\text{random}}\in A, & \text{otherwise} \end{array}\right.$$ (6)

where the heuristic function *H_t_* is derived from a heuristic policy *π^H^* that determines preferred actions over all the other actions, and *λ* is a coefficient to balance the value of predicted *Q* and heuristic function. In our cases, the heuristic policy is derived by maximizing current step reward, which is sensitive to the constraints and obstacles,

$$\pi^{H}=\underset{a}{\text{argmax}}\;r\left(s_{n},a\right)$$ (7)

Each feasible action is assigned a heuristic value in each iteration step to evaluate the action performance. In terms of Eq. 7, the value of the heuristic function *H_t_*(*s*, *π^H^*(*s*)) should be positive and higher than the variation among *Q*_1_(*s*, *a*) values so that the heuristic function can influence the choice of actions,

$$H_{t}(s,a)=\left\{\begin{array}{cc} {\text{max}}_{i}Q_{1}(s,a)-Q_{1}(s,a)+\beta , & a=\pi^{H}(s)\\ 0, & \text{otherwise} \end{array}\right.$$ (8)

where *β* is as small as possible to minimize the loss of the evaluate network. The action set in our case can be given as *A* = [*a*_1_, *a*_2_, *a*_3_, *a*_4_, *a*_5_, *a*_6_, *a*_7_, *a*_8_] at a specific state *s_t_*. Each action *a_i_* ∈ *A* corresponds to a value by calculating Eq. 7. Then, the *a_i_* that derives the biggest value is the heuristic policy, e.g., assume that the predicted *Q* value in a certain state *s_i_* is *Q*_1_ = [−100, −120, −110, −80, −90, −105, −115, −120], *β* = 1, and *π^H^*(*s*) = *a*_5_. Then, *H_t_*(*s_i_*, *a*_5_) = 11 can be calculated by Eq. 8, and zero for the other actions, which is *H* = [0, 0, 0, 0, 11, 0, 0, 0]. Substitute *H* into Eq. 7, then *Q*_1_ + *λH* = [−120, −110, −80, −90 + 11*λ*, −105, −115, −120]. If *λ* = 1, then the combined value of *Q*_1_ + *λH* associated with *a*_5_ (preferred action) is the biggest one compared to the other actions. The bigger the *λ*, the greater the influence of the heuristic function on action selection. Besides influencing the action selection in the learning process, *λ* is also working as a safety ensurance decided by a fuzzy logic rule that switches the planner from RL to the GHS method.

### Heuristically accelerated deep Q network (HADQN)

**Fig. 3.**
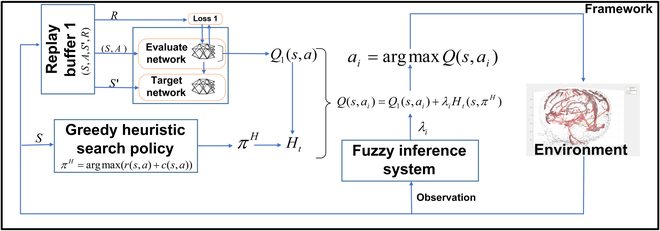
FL-HADQN framework for steerable needle-based neurosurgery path planning. By combining the heuristic function *H_t_* generated by policy *π^H^*, a *Q*(*s*, *a*) value is derived for action selection by the modified *ε* greedy algorithm. The fuzzy inference system is to adjust the coefficient of the heuristic function combination for a safer planning scheme.

*Q* value generation is affected by the current policy. Given the combined policy in the “Heuristics combination” section, the loss function of DQN is modified in this section. The heuristically accelerated RL algorithm (HARL) was firstly proposed by Bianchi et al. [23], which shows great potential in robotics. As an expansion of HARL, heuristically accelerated deep Q learning is proposed as a general RL algorithm that can be accelerated by heuristic functions. The training of vanilla DQN without a heuristic policy only depends on random exploration and exploitation, which leads to high computational costs in a neurosurgical environment. The heuristic function is commonly used in path planning algorithms to guide the agent toward the target. Combined with a heuristic function *H_t_*, the value-based RL algorithm can approximate the *Q* value function by neural networks together with *H_t_*, which can accelerate the exploration intuitively. Two neural networks (NNs), namely, the value evaluation network and target network, exactly the same as vanilla DQN, construct the HADQN. At the current state *s_j_*, the output $Q\left(s_{j},\pi_{j}^{c};\omega_{1}\right)$ of the value evaluation network with parameter *ω*_1_ is to evaluate the performance of the combined policy $\pi_{j}^{c}$, which is derived by the combined value of the heuristic function and *Q* value from the value evaluation network. The target network is for updating the target of the *j_th_* iteration:

$$y_{j}=r_{j}+\gamma \;\underset{\pi_{j+1}^{c}}{\text{max}}\hat{Q}(s_{j+1},\pi_{j+1}^{c};\omega_{2}^{j-1})$$ (9)

where $\omega_{2}^{j-1}$ is the parameter of the target network at *j* − 1*_th_* iteration. The loss function at *j_th_* iteration is given by,

$$L_{j}\left(\omega_{1}^{j},\omega_{2}^{j}\right)=E_{s,\pi ∼\rho }\left[{\left(y_{j}-Q\left(s_{j},\pi_{j}^{c};\omega_{2}\right)\right)}^{2}\right]$$ (10)

where the target is to minimize the target network output and evaluate network output. The HADQN framework is demonstrated and explained in Fig. 3 and Algorithm 1. In the *j_th_* iteration step, state *s_j_* of the steerable needle and reward *r_j_* are observed. Curvature and torsion of the steerable needle are constrained by the curve energy function during planning. Then, a (*S*, *A*, *S*^′^, *R*) transition pair observed from the environment is stored in a replay buffer. The state is transferred to the heuristic function *H*(*s_j_*) (decided by heuristic policy *π^H^*) as well. Once the replay buffer is filled up with the transition pairs, mini batches are selected as the training data of the NNs. Then, the predicted *Q* value *Q*_1_(*s*, *a*) from NNs and the heuristic value *H*(*s_n_*) from the heuristic function Ht jointly determine the action. The combined action is given by Eq. 6 following a modified epsilon-greedy policy. By repeating the above iteration process, the algorithm would find an optimal path in a much shorter period than the original DQN.

### Fuzzy inference system

To balance the *Q* value and the heuristic function value, *λ* in Eq. 6 should be selected properly. Instead of selecting *λ* by manual trial and error similar in in [22]. Fuzzy inference system (FIS) is used to evaluate *λ* adaptively in this section. FIS is an approach to variable processing for a problem that is ambiguous, as the concept of “cold” or “hot” for temperature and “close” or “far” for distance. FIS has been developed in decision-making and parameter tuning extensively. Wang et al. [24] proposed a FIS-based lane-changing behavior prediction algorithm that can reach 92.40% accuracy. Chen et al. [25] utilized a maximum defuzzification method to generate actions for steering angles or accelerating. A fuzzy parameter tuning scheme was proposed by Tan et al. [26] to control a hyper-redundant robot. FIS fulfills the role of *λ* selection in our planning scheme. Different *λ* has different influences on the heuristic combination. When applying a preoperative path planning algorithm for neurosurgery, safety criteria must take precedence to guarantee that the instrument has enough operating space. The risk-aware GHS algorithm can ensure the safest action toward the target in the current step, which is shown in Discussion. Thus, when the trajectory is close to the anatomical obstacles, GHS dominates in both the learning process and evaluating processes. On the basis of the parameters of the neurosurgery environment and the value of *λ*, a fuzzy inference system is proposed in our scheme. The distances from the needle to the target and the obstacles are selected as the inputs of the FIS.

**Fig. 4.**
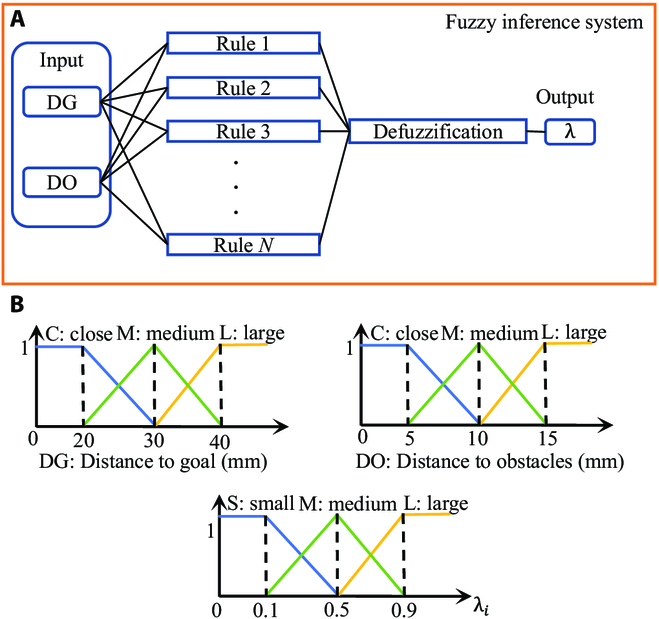
(A) Distance to the target and distance to the nearest obstacle are the inputs of the fuzzy inference system and are fuzzified by the fuzzy rules given in Table 1. *λ* is then defuzzificated by the weighted average method. (B) Membership functions of the fuzzy logic system.

Currently, there is still no risk quantification in neurosurgery. However, the consensus among surgeons is to keep the surgical trajectory away from blood vessels and some other anatomical obstacles as much as possible [27]. Furthermore, the threshold of insertion accuracy is 0.5 to 1.0 mm [27], which means that an improvement under 1.0 mm would be trivial. The threshold is used as a criterion in the experiments as well. On the basis of the judgment of the surgeon’s experience, as shown above, the input membership functions of FIS transform the specific values of distances to fuzzy values. The output membership functions represent the relationship between the safety criteria *λ* and the values processed by fuzzy rules. The fuzzy membership functions are shown in Fig. 4. Moreover, the fuzzy rules are shown in Table 1 as an **IF-THEN** format, where “DG” represents the distance from the needle tip to the target, and “DO” represents the distance from the needle tip to the nearest obstacle. The basic idea of the fuzzy rules is to speed up the training at the beginning stage (DG is large). In addition, as long as DO is close, then *λ* would be large so that the GHS algorithm can take over the system. When DG is medium, then we would like the DQN to take over so that the algorithm can explore the space for better solutions.

**Table 1.** Fuzzy rules.

**R**	**IF**		**THEN**
	**DG**	**DO**	***λ***
1	Close	Close	Large
2	Close	Medium	Medium
3	Close	Large	Small
4	Medium	Close	Small
5	Medium	Medium	Medium
6	Medium	Large	Large
7	Large	Close	Small
8	Large	Medium	Large
9	Large	Large	Large

Given a specific state *s_i_*, DG*_i_* and DO*_i_* can be calculated as well, e.g., DG*_i_* = 32 mm and DO*_i_* = 12 mm. The membership function in Fig. 4 can then calculate DG(Close) = 0, DG(Medium) = 0.8, DG(Large) = 0.2, DO(Close) = 0, DO(Medium) = 0.6, and DO(Large) = 0.4. Using the minimum rule, the output *λ*(Medium) =  Min (0.8,0.6) = 0.6, *λ*(Small) =  Min (0.8,0.4) = 0.4, *λ*(Medium) =  Min (0.2,0.6) = 0.2, *λ*(Large) =  Min (0.2,0.4) = 0.2 from rows 5 to 6 and rows 8 to 9 in Table 1.

After fuzzy logic transforms the input values into the membership degree of each set through fuzzification, several fire strength (FS) can be obtained through rules and operations. What we required is a specific value of *λ*; thus, a defuzzification process is included in the FIS as well. In this paper, we use the weighted average method, which is suitable for a symmetrical output membership function to defuzzificate the problem, given by,

$$\lambda =\frac{\sum_{i}FS_{i}\cdot OW_{i}}{\sum_{i}‍FS_{i}}$$ (11)

where OW*_i_* represents the output weight, OW(Small) = 0.2, OW(Medium) = 0.5, and OW(Large) = 0.8 under this condition. As calculated above FS_1_(Medium) = 0.6, FS_2_(Small) = 0.4, FS_3_(Medium) = 0.2, FS_4_(Large) = 0.2, and then *λ* = 0.457.

### Bezier curve interpolation

The generated raw zigzag-like movements are not suitable for guiding a steerable needle inside the brain tissue. Thus, a Bezier curve interpolation is considered to smooth the raw movements. The control points of the Bezier curve are the selected vertices calculated by Algorithm 1. Four points from $P_{0}^{i}$ to $P_{3}^{i}$ sequentially form a cubic Bezier curve. The *i_th_* segment ***B****_i_*(*t*) = [*x_i_*(*t*), *y_i_*(*t*), *z_i_*(*t*)]*^T^* of the whole curve is given by,

$$\begin{array}{c} B_{i}\left(t\right)={\left(1-t\right)}^{3}P_{0}^{i}+3t{\left(1-t\right)}^{2}P_{1}^{i}\\ +3t^{2}\left(1-t\right)P_{2}^{i}+t^{3}P_{3}^{i} \end{array}$$ (12)

Two consecutive curve connection requires *C*^2^ continuity for a smooth path generation. From Eq. 3, the curvature is a function of the first and second derivatives; thus, the *C*^2^ continuity conditions are set by,

$$\begin{array}{c} \underset{t\to 1}{\text{lim}}B_{i}\left(t\right)=\underset{t\to 0}{\text{lim}}B_{i+1}\left(t\right)\\ \underset{t\to 1}{\text{lim}}\frac{dB_{i}\left(t\right)}{\mathrm{dt}}=\underset{t\to 0}{\text{lim}}\frac{dB_{i+1}\left(t\right)}{\mathrm{dt}}\\ \underset{t\to 1}{\text{lim}}\frac{d^{2}B_{i}\left(t\right)}{dt^{2}}=\underset{t\to 0}{\text{lim}}\frac{d^{2}B_{i+1}\left(t\right)}{dt^{2}}. \end{array}$$ (13)

The relation of the 2 consecutive curves control points can be derived by solving Eq. 12,

$$\begin{array}{c} P_{0}^{i+1}=P_{3}^{i}\\ P_{1}^{i+1}=2P_{3}^{i}-P_{2}^{i}\\ P_{2}^{i+1}=4P_{3}^{i}-4P_{2}^{i}+P_{1}^{i}. \end{array}$$ (14)

The original control points generated by Algorithm 1 would cause sharp edges when joining 2 Bezier curves. In the (*i* + 1)*_th_* Bezier curve ***B***_*i* + 1_(*t*), $P_{0}^{i+1}$ coincides with $P_{3}^{i}$. $P_{1}^{i+1}$ and $P_{2}^{i+1}$ calculated by Algorithm 1 would be mutated by Eq. 13 for *C*^2^ continuity. Then, $P_{3}^{i+1}$ is decided by Algorithm 1 to define the direction and final position of the curve.

## Results

### Simulation setup and environment description

**Fig. 5.**
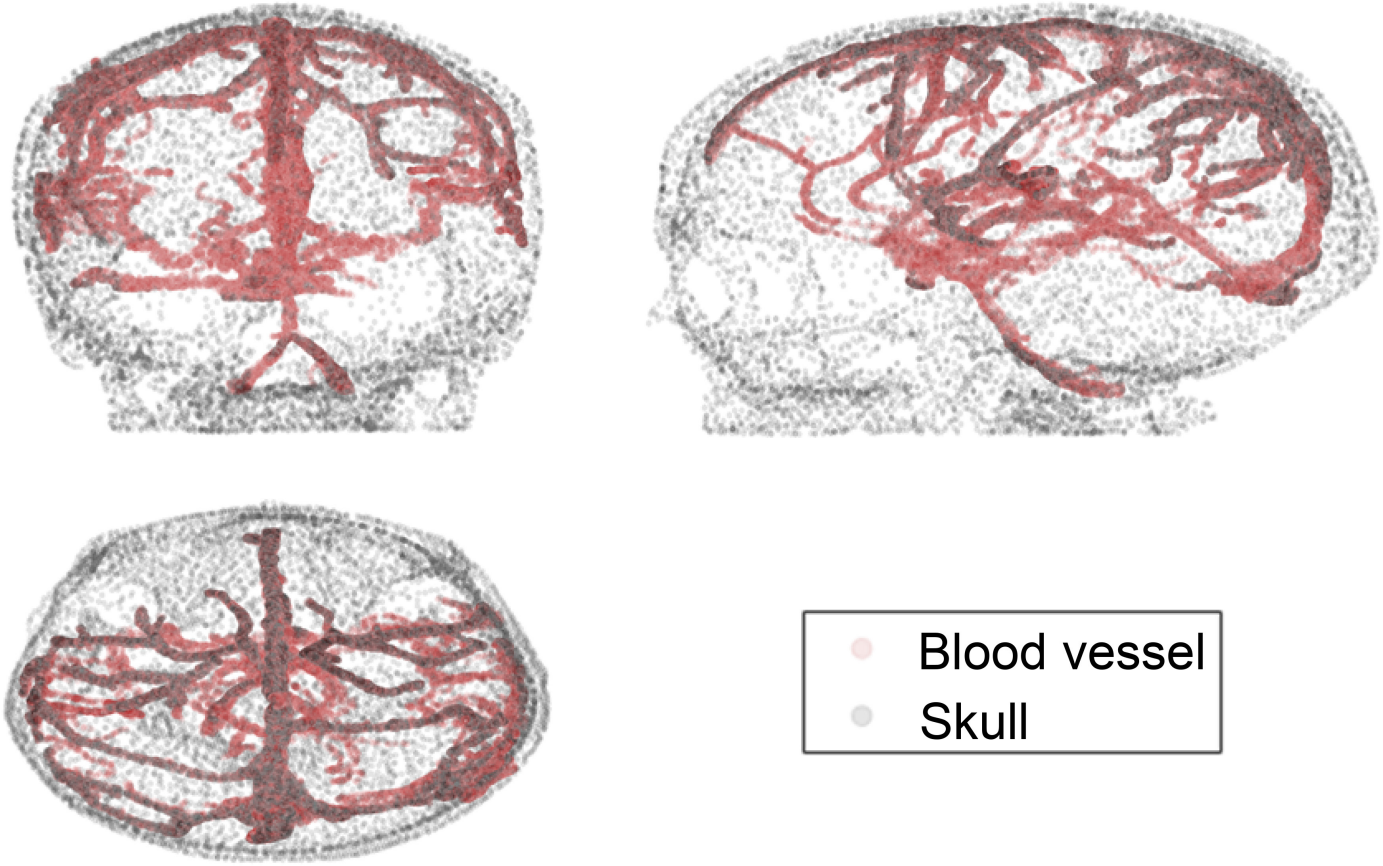
Planning environment of the proposed algorithm in this paper. Blood vessels and the skull are composed of red points and gray points, respectively.

To build a training environment, the original medical imaging data are acquired from The Cancer Imaging Archive [28], which is funded by the Cancer Imaging Program, which provided authorization to the high-resolution MRI DICOM dataset [29] for supporting the research. DICOM is the standard file format for radiological imaging and can be read and visualized by several software, as 3D Slicer [30]. Using 3D Slicer, brain blood vessels were segmented and exported as STL files that consist of the vertices of skulls, blood vessels that form the planning environment. The extracted environment is shown in Fig. 5.

Twenty pairs of entry points and target points are selected to test the effectiveness of the proposed FL-HADQN algorithm after training. The superiority of an effective and safety-aware preoperative path planner employing FL-HADQN over traditional GHS algorithm and original DQN algorithm from training time costs, maximum curvature, minimum distance to anatomical obstacles, and average distance to anatomical obstacles, 4 aspects, is presented in the following experiments. All tests were performed using Python 3, on a Windows machine (Win 11 21H2, 12th Gen Intel(R) Core(TM) i7-12700H CPU @ 2.30 GHz, 32 GB of RAM).

### Training of the proposed FL-HADQN

RL algorithms always suffer from low training efficiency, resulting in tedious training processes. The proposed FL-HADQN solves this problem by combining a heuristic function derived from a greedy heuristic policy. The training target is to find a safe path from entry points to target points with minimum curvature as well as obstacle avoidance.

In FL-HADQN, 2 fully connected neural networks with the same structure (3 hidden layers, each with 180 nodes) are used, namely, target network and value evaluation network. The input and output of the fully connected neural networks are (*x*_tar_ − *x_i_*, *y*_tar_ − *y_i_*, *z*_tar_ − *z_i_*) and 8 different *Q* values corresponding to 8 actions, respectively. For the action selection stage, a modified epsilon-greedy policy is shown as Eq. 6. *ε* starts to decay from 1 with a rate of 0.995 during exploration. Once the randomly generated number *p* ∈ [0, 1] is bigger than *ε*, then the action can be selected by argmax*_a_*(*Q*_1_(*s*, *a*) + *λH_t_*(*s*, *a*)), otherwise the action would be chosen randomly. It should be noted that if *λ* = 1 during training, then the algorithm is in HADQN mode, while if *λ* is selected by a fuzzy inference system, then the algorithm is in FL-HADQN mode. With a learning rate of 0.0001, the Adam optimizer optimizes a gradient descent temporal difference error step for training the data after the replay buffer is filled up by state exploration. Each episode has 250 iteration steps. The episode would be terminated if the agent reached the target or the iteration steps run out. If the curvature in one step exceeded the maximum curvature constraint *k*_max_ or the needle tip contact with the anatomical obstacles, then this episode would be terminated as well and a big negative reward is transferred to the agent. All the hyperparameters are summarized in Table 2.

**Table 2.** Hyper parameters.

Discount factor *γ*	0.95
Replay buffer size	10,000
Learning rate *α*	0.001
Batch size	100
Max episodes	150
Initial *ε*	1.0
*ε* decay rate	0.9995
Target network update frequency	200

**Fig. 6.**
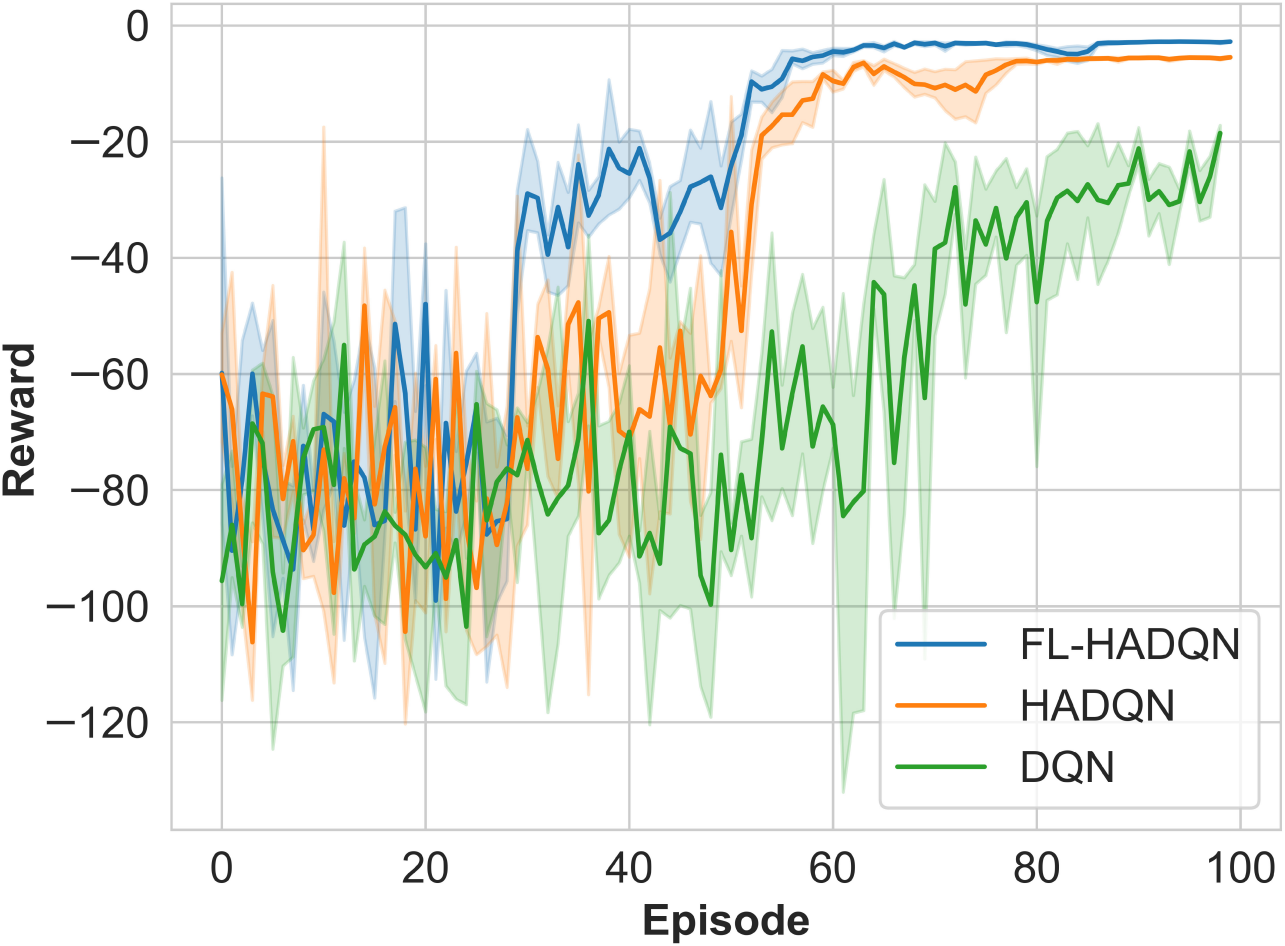
Training processes of FL-HADQN, HADQN, and DQN.

The accumulated rewards illustrate the performance of the algorithms and are usually covered to a high value if the agent successfully reaches the target. In Fig. 6, it is obvious that FL-HADQN and HADQN spend far less training time than the original DQN. FL-HADQN can be converged around 60 episodes, however, far more than 100 episodes for DQN. From the DQN reward plot, the reward vibrates greatly around 60 to 80 episodes and hardly can be converged before 100 episodes. Furthermore, the accumulated reward of FL-HADQN is higher than the original DQN at the end of the training, because the heuristic policy takes into account the trajectories’ curvature constraint, resulting in shorter trajectories with smaller curvatures than DQN.

**Fig. 7.**
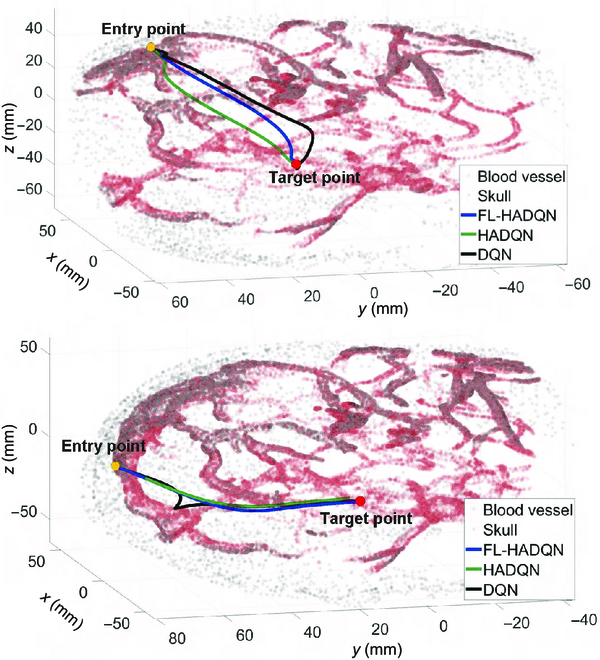
Two groups of trajectories with different entry points and target points. The black curves generated by DQN exist as excessive curvatures during planning.

### Evaluation

After training, FL-HADQN, HADQN, and DQN were commanded to plan the paths from 20 different groups of entry points and target points deep inside the brain. Because of the generalization problem in RL and heuristic function setup in the GHS algorithm, the 3 algorithms might fail to reach the target in some cases. Considering of the diameter of 1 mm of the steerable, if the minimum distance is smaller than 1 mm in one case, then the corresponding path would be recorded as a fail as well. The success rate of FL-HADQN, HADQN, DQN, and the GHS algorithm is 95%, 95%, 75%, and 90%, respectively, among the 20 trajectories.

The first test aimed to find out the effects of curvature and torsion energy constraints on the generated paths.

**Fig. 8.**
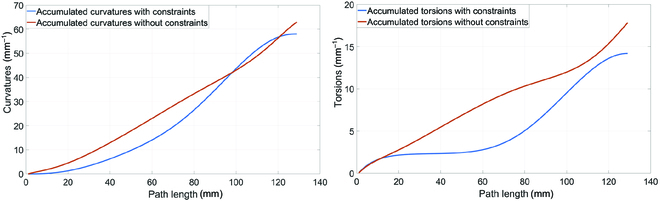
Accumulated curvatures and torsions comparisons between constrained algorithm and unconstrained algorithm.

**Fig. 9.**
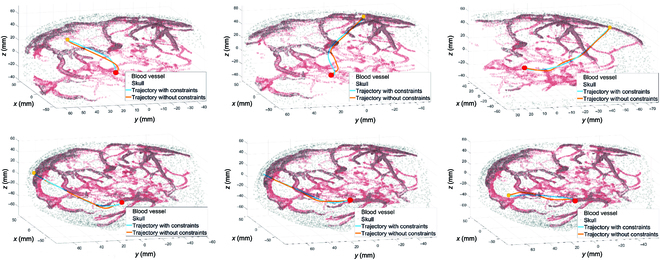
Different trajectories with curvature and torsion constraints or without constraints. The trajectories with constraints can reach the targets with smaller curvatures and torsions than trajectories without constraints. The yellow points and red points are the entry points and target points, respectively.

The results are given in Fig. 8, where accumulated curvatures and torsions generated by constrained algorithms are far smaller than unconstrained ones most of the time. The sampled paths, as shown in Fig. 9, intuitively demonstrate that the energy-constrained algorithm produced smoother and smaller curvature results than the algorithm without constraints.

**Fig. 10.**
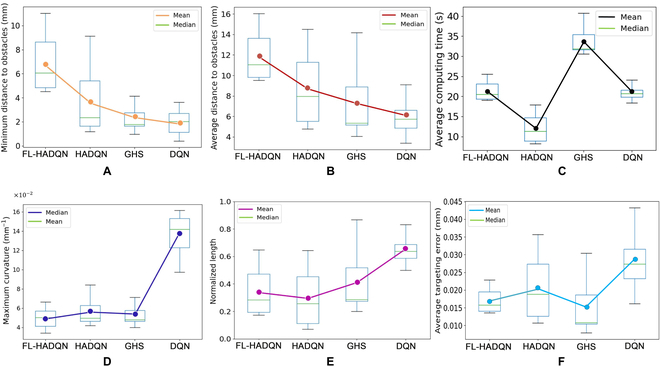
Twenty trajectory test results using FL-HADQN, HADQN, GHS algorithm, and DQN. (A) *d*_min_ comparisons. (B) $\bar{d}$ comparisons. (C) $\bar{t}$ comparisons. (D) *k*_max_ comparisons. (E) $\bar{l}$ comparisons. (F) $\bar{e}$ comparisons.

The second test was among the 3 learning based-algorithms: FL-HADQN, HADQN, and DQN [17] to compare their normalized path lengths, the minimum distance to obstacles, the average distance to obstacles, and the maximum curvature, 4 aspects when commanding the 20 trajectories. The mean values of each indicator are given in both Table 3 and Fig. 10.

**Table 3.** Indicator comparisons among the learning-based algorithms and the greedy algorithm.

	GHS	RRT	FL-HADQN	HADQN	DQN
$\bar{l}$ (normalized)	0.39	0.92	0.35	0.30	0.61
*d*_min_ (mm)	2.58	4.33	6.80	3.82	1.92
$\bar{d}\left(\mathrm{mm}\right)$	7.42	8.05	11.54	8.44	6.10
*k*_max_ (mm^−1^)	0.052	0.162	0.046	0.058	0.139
$\bar{t}\left(s\right)$	34.25	19.54	22.32	12.42	20.64
$\bar{e}\left(\mathrm{mm}\right)$	0.015	0.015	0.017	0.022	0.028
Segments	3.2	8.9	4.3	3.4	6.5

#### The minimum distance to obstacles (**d**_**min**_)

The minimum distance to anatomical obstacles is one of the most important safety criteria for path planners in neurosurgeries. Comparing the tested 20 trajectories, the minimum distance to obstacle results are shown in the boxplot in Fig. 10A. With the combination of a heuristic function, FL-HADQN takes advantage of efficient exploration and a fuzzy logic safety ensurance. The minimum distance to obstacles for FL-HADQN, HADQN, and DQN is decreasing. Moreover, *d*_min_ of each trajectory appears in the last few iteration steps that will not be recorded as data because the target is near the obstacle. In this *d*_min_ boxplot, the median and 75 percentile of FL-HADQN is over 6 mm, however, only around 2.5 mm for HADQN and DQN, which means that the obstacle-avoiding ability by using varying *λ* generated by the fuzzy inference system in FL-DQN outperforms HADQN and DQN. In addition, the minimum value of the GHS boxplot is higher than HADQN, which proves that GHS tends to keep away from obstacles; thus, the proposal of the fuzzy rules above is correct.

#### The average distance to obstacles ($\bar{d}$)

The minimum distance to obstacles can not be the only criterion of distance to obstacles, e.g., if *d*_min_ = 1.2 mm, and most segments’ distances to obstacles are from 1.2 to 1.5 mm; the trajectory would be categorized as a bad path as well, because most parts of the path are too close to the obstacles for a needle insertion process with the insertion accuracy threshold of around 1 mm. The mean value of $\bar{d}$ for FL-HADQN, HADQN, GHS, and DQN shown in Fig. 10B is 11.86, 8.87, 7.82, and 6.64 mm, respectively.

#### The average computing time ($\bar{t}$)

We test the execution time after training for all 3 RL-based algorithms. Because all the parameters for FL-HADQN, HADQN, and DQN are stored in H5 files, the only calculation required is to call the NNs stored. Thus, the average execution time for FL-HADQN, HADQN, and DQN are 21.40, 12.43, and 20.92 s, respectively, and can be found in Fig. 10C.

#### The maximum curvature (***k***_**max**_)

Needle curvature is also considered to be one of the most important safety metrics [7] in steerable needle-based surgery. In this paper, the curvature of a path is constrained by the curve energy function to find the minimal energy curve [31]. The planned trajectory samples with Bezier curve interpolation are illustrated in Fig. 7, which shows the black-colored trajectories planned by DQN that sometimes would deviate from the other trajectories apparently and results in an unbearable curvature that might damage brain tissues because of the DQN generalization problem. Different paths’ curvatures generated by the 3 algorithms are compared in Fig. 10D, which illustrates that the maximum curvature of the curve produced by DQN is up to 0.162 mm^−1^. Compared to DQN, the blue-colored trajectories planned by FL-HADQN end with smoother and smaller curvature curves.

#### The normalized paths length ($\bar{l}$)

The trajectory length of a path planning problem is the priority no matter in the mobile robots field or the needle insertion process. The shorter the path, the less risk of damage is to the brain tissue. In Fig. 10E, $\bar{l}$ of HADQN is the smallest, and for FL-HADQN, $\bar{l}$ is slightly larger than HADQN because of the deviation given by the fuzzy inference system when approaching obstacles, which is trivial because the difference is pretty small.

#### The average targeting error ($\bar{e}$)

The absolute average targeting error in Fig. 10F shows the planned trajectories whether reaching the target or not. To make RL-based algorithms terminate successfully, we relaxed the threshold appropriately. When the absolute targeting error $\bar{e}<0.05\;\mathrm{mm}$, the process would be terminated. $\bar{e}$ for FL-HADQN, HADQN, and DQN are 0.017, 0.022, and 0.028 mm, respectively.

#### The average number of curved segments

The number of curved segments generated by each algorithm is also shown in Table 3. This indicator shows how many turns there are when inserting a needle along the planned trajectories. RRT generated more path curve segments than the other algorithms because of the random sampling. GHS- and HARL-based planners generate less curved segments because of the heuristics function attracting the agent to reach the target. The results are given in Table 3 as well.

The third test was conducted to compare the performance of the 3 RL-based algorithms with respect to the GHS algorithm and RRT-based planning algorithm. The heuristic function is set to be *f*(*n*) = *g*(*n*) + *h*(*n*) + *o*(*n*). Usually *g*(*n*) is a function of Euclidean distance or Manhattan distance between the needle tip and the entry point. In this paper, however, *g*(*n*) represents the curve energy instead given by Eq. 2 to be consistent with the proposed algorithm, which is in the same functionality to use Euclidean distance and Manhattan distance. *h*(*n*) still represents the Euclidean distance between the needle tip and target point, and *o*(*n*) is for obstacle avoidance used in the proposed algorithm. The RRT random sampling probability was 0.5. Both tests were run on a pair of entry and target points. The comparisons of GHS and RRT include path lengths, minimum distance to obstacles, and average distance to obstacles that were given in Table 3 as well.

## Discussion

In this paper, we presented the superiority of a safe path planning algorithm using the proposed FL-HADQN framework compared to traditional graph-based and original DQN-based path planning algorithms. The combination of a heuristic function greatly improves the training efficiency. The integration of a fuzzy inference system and a curve energy-constrained reward function guarantees the obstacles' avoiding ability with minimum curvatures and minimum torsions of the planned path. The proposed framework demonstrates the potential of adopting RL-based path planning algorithm with a good balance of efficiency and efficacy. To further improve the generalization ability for different brain maps, in the next step, we will make the distance to obstacles as a state in RL and take the intraoperative local tissue deformation into account, so that the algorithm can be extended from offline preoperative planning to online intraoperative planning and adjustment.

## Data Availability

The data used to support the findings of this study are included within the article.

## Supplementary Materials

Supplementary 1. The brain environment data. Supplementary 2. The path planning code.
